# Mechanisms of Ghrelin Anti-Heart Failure: Inhibition of Ang II-Induced Cardiomyocyte Apoptosis by Down-Regulating AT1R Expression

**DOI:** 10.1371/journal.pone.0085785

**Published:** 2014-01-21

**Authors:** Chunyan Yang, Zhonghui Liu, Kai Liu, Ping Yang

**Affiliations:** 1 Department of Cardiology, China-Japan Union Hospital, Jilin University, Changchun, China; 2 Department of Immunology, Norman Bethune College of Medicine, Jilin University, Changchun, China; 3 Department of Hepatobiliary and Pancreatic Surgery, First Hospital, Jilin University, Changchun, China; University of Otago, New Zealand

## Abstract

**Background:**

Ghrelin is a novel growth hormone–releasing peptide administered to treat chronic heart failure (CHF). However, the underlying mechanism of its protective effects against heart failure (HF) remains unclear.

**Methods and Results:**

A total of 68 patients with CHF and 20 healthy individuals were included. The serum levels of Angiotensin II (Ang II) and ghrelin were measured using ELISA. The results showed that Ang II and ghrelin were both significantly increased in CHF patients and that the ghrelin levels were significantly positively correlated with Ang II. The left anterior descending coronary artery was ligated to establish a rat model of CHF, and cultured cardiomyocytes from neonatal rats were stimulated with Ang II to explore the role of ghrelin in CHF. The results showed that ghrelin inhibited cardiomyocyte apoptosis both in vivo and in vitro. Furthermore, caspase-3 expression was examined, and the results revealed that Ang II induces cardiomyocyte apoptosis through the caspase-3 pathway, whereas ghrelin inhibits this action. Lastly, to further elucidate the mechanism by which ghrelin inhibits Ang II action, the expression of the AT1 and AT2 receptors was evaluated; the results showed that Ang II up-regulates the AT1 and AT2 receptors in cardiomyocytes, whereas ghrelin inhibits AT1 receptor up-regulation but does not affect AT2 receptor expression.

**Conclusions:**

These data suggest that the serum levels of ghrelin are significantly positively correlated with Ang II in CHF patients and that ghrelin can inhibit Ang II-induced cardiomyocyte apoptosis by down-regulating AT1R, thereby playing a role in preventing HF.

## Introduction

Chronic heart failure (CHF) is the ultimate outcome of most cardiovascular diseases and is a major cause of disability and death in cardiovascular disease patients [1], [2]. Although CHF has many causes, a gradual decrease in cardiomyocyte number is one of the most important contributing factors [3], [4]. Increasing evidence indicates that one of the important forms of cardiomyocyte loss during CHF is cardiomyocyte apoptosis, which can be lethal even at very low levels [5]–[7]. Therefore, limiting cardiac muscle loss by inhibiting cardiomyocyte apoptosis may have implications for heart failure (HF) treatment.

Experimental studies of HF in animal models and patients suggest that the cardiac renin-angiotensin system (RAS) is activated and that angiotensin II (Ang II) production is enhanced [8]. Ang II regulates cardiac contractility, cell communication, impulse propagation, cardiac remodeling, growth, and apoptosis by activating the Ang II type 1 (AT1) and type 2 (AT2) receptors [9], [10], which are present in the adult rat ventricular myocardium; the AT1 receptor accounts for approximately 50–70% of specific Ang II binding [11], [12]. Recent evidence has suggested that most known effects of Ang II in the cardiovascular system are mediated by the AT1 receptor. Moreover, some studies have also reported that the AT_2_ receptor is involved in inhibition of cellular differentiation, growth, and apoptosis [13]. However, the role of the angiotensin AT1 and AT2 receptors in inducing cardiomyocyte apoptosis remains unclear [14]–[16].

The growth hormone–releasing peptide ghrelin is a novel, 28-amino acid peptide that was isolated from the stomach in 1999 [17]. Ghrelin is an endogenous ligand of the growth hormone secretagogue receptor (GHS-R) and has several biological activities, including the stimulation of GH secretion and promotion of food intake, which has been linked to obesity [18]. Some studies have reported that ghrelin confers a variety of potentially beneficial cardiovascular effects, which include reducing blood pressure, increasing cardiac contractility, protecting endothelial cells, improving myocardial energy metabolism, regulating atherosclerosis, preventing ischemia/reperfusion injury, and improving the prognoses of myocardial infarction (MI) and HF [19]–[22].

Our previous studies found that ghrelin protected H9c2 cardiomyocytes from Ang II-induced cell death [22]. However, the H9c2 cell line is not a “real” cardiomyocyte. Although H9c2 cells conserve the biological features of myocytes, they are not terminally differentiated cardiomyocytes and do not possess organized sarcomeres. H9c2 cells have both cardiomyocyte and skeletal muscle properties and seem to become more dedifferentiated with each subsequent cell passage. With the exponential division of H9c2 cells, myoblast fusion was not found, thereby indicating differentiation toward a skeletal muscle cell-like phenotype [23]. Therefore, determining whether ghrelin exerts an antiapoptotic effect on cardiomyocytes in vivo and in vitro is necessary. In the present study, we analyzed the relationship between ghrelin and HF by measuring ghrelin levels in the peripheral blood of patients with HF. Furthermore, we verified the relationship between ghrelin and HF in a rat model of HF. We also cultured primary neonatal rat cardiomyocytes and investigated the effects of ghrelin on Ang II-induced cardiomyocyte apoptosis and the underlying cellular and molecular mechanisms by assessing AT1R and AT2R. The aim of this study was to provide an experimental basis for the use of ghrelin to treat HF in a clinical setting.

## Materials and Methods

### Ethics statement

The study was approved by the Ethics Committee of China-Japan Union Hospital of Jilin University, China. All subjects provided signed informed consent prior to entering the study.

### Patients

The study population comprised 68 patients with CHF on admission (mean age 55±11 years; 32 males and 36 females) classified into 3 groups according to the New York Heart Association Functional Classification: NYHA II (11 cases), NYHA III (26 cases), and NYHA IV (31 cases). All patients' cardiac diagnoses were made on the basis of clinical examination, laboratory investigations, electrocardiography, and echocardiography; patients with lung, liver, renal, hematological, or other comorbidities were excluded. The study also included 20 healthy individuals as a control group (mean age 53±7 years; 9 males and 11 females). Informed consent was obtained from the parents of the subjects. Specimen collection was performed between 6 and 7 a.m. by venipuncture. Three milliliters of blood was allowed to clot at room temperature for 60 minutes. The serum was separated by centrifugation at 1600×*g* for 10 minutes at 4°C and then stored at −80°C. The ethics committee of the National Cardiovascular Center approved the study, and all patients gave written informed consent.

### Rat model of HF

Female Wistar rats with a body weight of 200 to 220 g were obtained from the Center for Laboratory Animals, Medical College, Jilin University. To establish the HF model, post-MI, diethyl ether-anesthetized rats were fixed on an operating table. The thoracic cavity was opened to expose the heart, and the left anterior descending (LAD) coronary arteries were ligated. In the control group, rats underwent the same procedure, except the suture under the coronary artery was left untied (*n* = 10). Then, the heart was returned to its normal position, and the thorax was immediately closed. All rats were fed a standard diet and tap water and maintained at a room temperature of 21°C±1°C under a 12-hour light/dark cycle for 4 weeks. Then, the MI rats were randomly divided into 2 groups: one received synthetic rat ghrelin (*n* = 10), and the other received saline (*n* = 10). Ghrelin (100 µg/kg twice daily, the dose shown to improve LV function in rats with CHF) or saline was administered subcutaneously for 4 weeks. All experimental procedures were approved by the Animal Ethics Committee of Jilin University.

### Hemodynamic measurements

The rats were anesthetized via the intraperitoneal injection of 3% pentobarbital sodium (30 mg/kg). A catheter was inserted into the right carotid artery and then further advanced into the left ventricular chamber to record the left ventricular systolic pressure (LVSP), left ventricular end-diastolic pressure (LVEDP), and rate of contraction and relaxation (±dp/dt). The systolic blood pressure (SBP), diastolic blood pressure (DBP), and heart rate (HR) were also recorded by an 8-channel polygraph system (RM-6000). To measure hemodynamic parameters, the rats from each group were weighed and subsequently anesthetized by an intraperitoneal injection of 30 mg/kg 3% pentobarbital sodium. The right common carotid artery was isolated, left ventricular cannulation was performed, and the cannula was connected to a pressure transducer and an AP-621G carrier amplifier. The LVSP and LVEDP were recorded using an RM-6000 8-channel polygraph system. LVSP electrical signals were inputted into a differentiator to plot the maximum rate of left ventricular pressure increase and decrease (±dp/dt_max_).

### Hematoxylin and eosin staining

The noninfarcted left ventricular tissues were fixed in 4% paraformaldehyde, embedded in paraffin, and sliced into 5-µm sections. The myocardium sections (5 µm) were stained with hematoxylin and eosin (H&E) to analyze the morphological changes in the myocardium.

### Primary culture of neonatal rat cardiomyocytes

Primary cultures of neonatal rat cardiomyocytes were prepared from the cardiac ventricles of neonatal Wistar rats as described previously. Briefly, cardiac ventricles taken from 1- to 2-day-old Wistar rat neonates were gently minced and enzymatically dissociated using trypsin in 0.02 mol/L phosphate-buffered saline (PBS), pH 7.4, at 37°C. The dissociated cells were then filtered through 200-µm mesh, collected by centrifugation, and incubated for 1.5 hours at 37°C in a cell culture flask. The non-cardiomyocytes, fibroblasts or endothelial cells, can attach more easily to plastic surfaces than the cardiomyocytes. After 1.5 hours, the supernatant was collected and plated at a density of 5×10^5^/mL on a 60-mm dish in DMEM/F-12 culture medium containing 10% fetal calf serum and antibiotics (100 U/mL penicillin and 100 mg/mL streptomycin) for 48 hours. Simultaneously, 5-bromo-2′-deoxyuridine (BrdU, 10^−4^ mol/L) was added to prevent the proliferation of non-myocytes. After 48 hours, the cells were seeded and serum-deprived for 24 hours. Then, the cells were divided into the following groups: ghrelin (0.1 µmol/L), Ang II (0.1 µmol/L), both agents (0.1 µmol/L ghrelin + 0.1 µmol/L Ang II), or culture medium. These solutions were each added to the culture dishes for 24 hours.

### Immunohistochemical staining

Immunohistochemical staining for caspase-3 and AT1R was performed on the myocardial tissue and cardiomyocytes in vitro. The noninfarcted left ventricular tissue was deparaffinized, rehydrated in a graded series of alcohol solutions, and washed twice in distilled water. The sections were incubated with endogenous peroxidase blocked in 50 µL of 3% H_2_O_2_ at room temperature for 10 minutes and then washed with PBS (pH 7.4) 3 times for 3 minutes each. After incubating in 2% BSA in PBS at room temperature for 30 min, the sections were washed once with PBS. The caspase-3 or AT1R antibodies were added, and the sections were incubated at 4°C overnight. The caspase-3 and AT1R proteins were assayed with an Ultrasensitive SP kit. The sections were counterstained with hematoxylin. The incubation of the tissue sections with normal rabbit IgG served as a negative control. In a separate procedure, cardiomyocytes were seeded on slides, incubated for 24 hours, and then washed 3 times with PBS for 5 minutes each. The cells were also incubated with 3% H_2_O_2_ at room temperature for 10 minutes, followed by the remaining steps as described above.

### Agarose gel electrophoresis for DNA fragmentation

Cardiomyocytes (1−2×10^6^) were washed with PBS and pelleted by centrifugation. The cell pellets were then re-suspended in 500 µl of protein lysis buffer (75 mmol/L NaCl, 0.5% sodium dodecyl sulfate, 10 mmol/L Tris-HCl, and 10 mmol/L EDTA, pH 8.0) containing 200 µg/mL RNase (Sigma-Aldrich) and incubated at 37°C for 1 hour. The cell solution was then combined with 150 µg/mL proteinase K, incubated at 50°C for 3 hours, and subjected to 3 rounds of phenol-chloroform DNA extraction. The DNA was then precipitated using 100% cold ethanol and ammonium salt. Then, the DNA was collected by centrifugation and dissolved in TE buffer (10 mmol/L Tris-HCl, 1 mmol/L EDTA, pH 8.0). The DNA concentration was determined, and 10 µg of each DNA sample was electrophoresed on a 1.5% agarose gel and photographed.

### TUNEL assay

A terminal deoxynucleotidyl transferase-mediated dUTP-biotin nick end-labeling (TUNEL) assay was performed on cells that were plated on glass coverslips using an in situ apoptosis detection kit according to the manufacturer's instructions. Briefly, after stimulation for 24 hours with 0.1 µmol/L ghrelin and 0.1 µmol/L Ang II, the cells were washed 3 times with 0.01 mol/L PBS, fixed in 4% paraformaldehyde for 1 hour, washed another 3 times with PBS, and incubated with permeabilizing solution (0.1% Triton X-100 and 0.1% sodium citrate) on ice for 2 minutes. After 3 washes, the liquid around the samples was drained. The samples were then incubated with the TUNEL reagent at 37°C for 1 hour in a dark, humid chamber. Permeabilized cells that were incubated with 2000 U of DNase I (1 mg/mL, Sigma) in 50 mmol/L Tris-HCl (pH 7.5), 10 mmol/L MgCl_2_, and 1 mg/mL BSA were used as a positive control. As a negative control, the cells were treated with the labeling solution alone. After 3 washes with PBS, the samples were visualized and photographed under a fluorescence microscope (excitation wavelength: 450–500 nm; emission wavelength: 515–565 nm, green color). Finally, the apoptosis rates were calculated.

### Quantitative Real-Time RT-PCR

Real-time quantitative PCR was performed as described previously [19]. Briefly, the total RNA was extracted with TRIzol Reagent. The RNA concentrations and purity were determined using a spectrophotometer. A total of 200 ng of RNA was reverse-transcribed into cDNA. Real-time fluorescent quantitative PCR was performed with the cDNA using the SYBR® RT-PCR Kit and the ABI 7300 Real-Time PCR System (Applied Biosystems) according to the manufacturers' instructions. The PCR was performed as follows: 1 cycle of 95°C for 10 s; 40 cycles of 95°C for 5 s and 60°C for 31 s (fluorescence was collected at this stage); and 1 cycle of 95°C for 15 s, 60°C for 30 s, and 95°C for 15 s. The standard curve was plotted, and quantitative PCR analysis was performed according to the standard curve. The primers used for qRT-PCR (Table 1) were synthesized by Sangon Biotech (Shanghai, China).

**Table 1:** Primer sequences used in real-time PCR.

Target	GenBank	Primer	Sequences
GAPDH	X02231.1	Sense	5′-CATCACCATCTTCCAGGAGCG-3′
		Antisense	5′-TGACCTTGCCCACAGCCTTG-3′
AT1	M87003.1	Sense	5′-GCCAAAGTCACCTGCATCAT-3′
		Antisense	5′-AATTTTTTCCCCAGAAAGCC-3′
AT2	BC161802.1	Sense	5′-TGAGTCCGCATTTAACTGC-3′
		Antisense	5′-ACCACTGAGCATATTTCTCGGG-3′
Caspase-3	NM_012922.2	Sense	5′-CATGACCCGTCCCTTGAA-3′
		Antisense	5′-CCGACTTCCTGTATGCTTACTCTA-3′

### Western blot

Total protein from cardiomyocytes was extracted and separated by sodium dodecyl sulfate-polyacrylamide gel electrophoresis (SDS-PAGE). The proteins were then transferred to a polyvinylidene difluoride (PVDF) membrane, blocked, and probed with primary antibodies against AT1R and GAPDH. Next, the membrane was incubated with horseradish peroxidase-conjugated goat anti-rabbit IgG. Finally, the bound antibody complexes were detected using a chemiluminescence reagent (ECL-Plus; Amersham Pharmacia Biotech).

### Statistics

The data are reported as the means ± SD. Comparisons were made between different treatment groups using ANOVA followed by the Dunnett post hoc test for differences. The data for percent changes were analyzed using the Kruskal-Wallis *H-test*. A value of *P*<0.05 was considered statistically significant. All experiments conformed to the Chinese Academy of Medical Sciences ethics code of practice.

## Results

### Ghrelin and Ang II were upregulated in patients with CHF, and ghrelin levels were positively correlated with Ang II

Demographic information for the healthy controls and HF patients is shown in Table 2. There were no significant differences between the groups in terms of mean age or sex (both P>0.05).

**Table 2:** Characteristics of patients with CHF and the control group.

	Control (n = 20)	CHF (n = 68)
**Age, y**	53±7	55±11
**Sex, male/female**	9/11	32/36
**NYHA functional class, n**		
**I**	20	
**II**		11
**III**		26
**IV**		31

**Figure 1:**
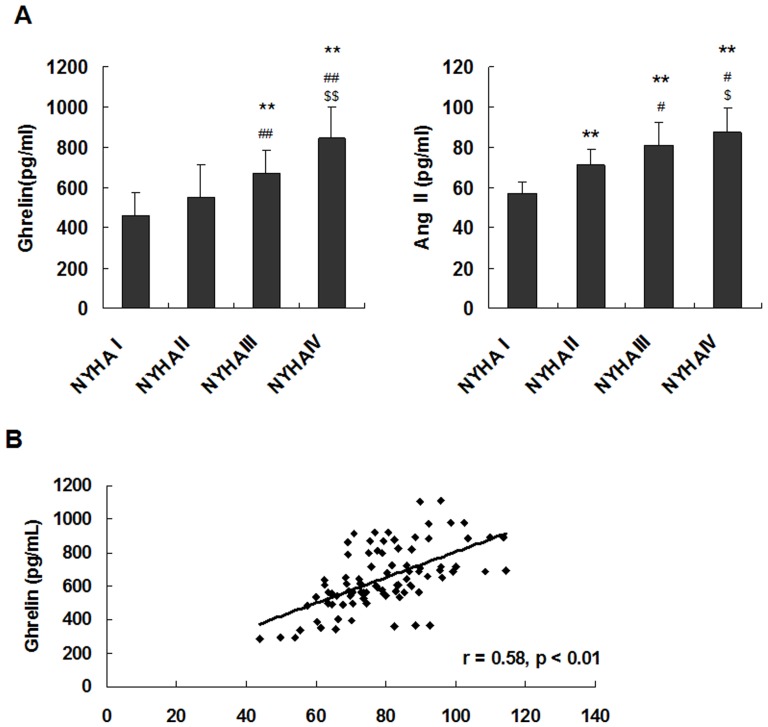
Ghrelin and Ang II were upregulated in patients with CHF. (A) Serum levels of ghrelin and Ang II in patients with CHF and in healthy subjects. (B) Correlation of ghrelin with Ang II in patients with CHF and in healthy subjects. The data are presented as the means ± SD. **P<0.01 vs. healthy control subjects (NYHAI); ^#^P<0.05, ^##^P<0.01 vs. NYHA II; ^$^P<0.05, ^$$^P<0.01 vs. NYHA III.

Serum Ang II levels were significantly higher in the patients with CHF, and the expression of ghrelin was positively correlated with the severity of CHF. Serum Ang II levels were significantly higher in the patients with CHF (NYHA II, NYHA III, and NYHA IV) than in the healthy controls (P<0.05), higher in the NYHA III and NYHA IV groups than in the NYHA II group (P<0.05), and higher in the NYHA IV group than in the NYHA III group (P<0.05). Similarly, serum ghrelin levels were significantly increased in the patients with CHF. Serum ghrelin levels were significantly higher in the patients with CHF (NYHA III and NYHA IV) than in the healthy controls (P<0.01) and were significantly higher in the patients with NYHA class IV CHF than in the patients with NYHA class III CHF (P<0.01) (Fig. 1A).

Furthermore, the study showed that serum ghrelin correlated significantly with serum Ang II (*r* = 0.58, P<0.01) (Fig. 1B). These results suggested that ghrelin may play a role in preventing HF by inhibiting Ang II.

### The effect of ghrelin on HF

**Figure 2:**
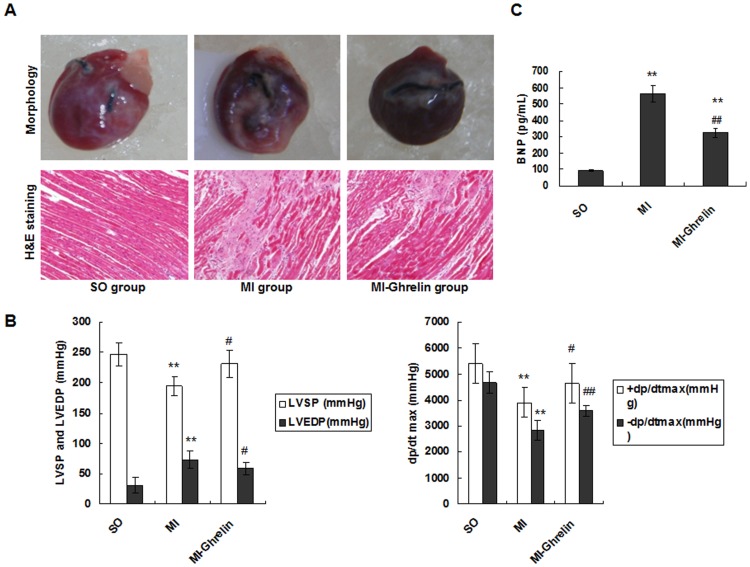
The effect of ghrelin on HF. (A) Representative images of hearts. Four weeks after ghrelin administration, the heart was removed, and the tissue sections were stained with H&E (×200 magnification). (B) Hemodynamic index, including ±dp/dt_max_, LVEDP, and LVSP. +dp/dt_max_, maximal rate of the rise in blood pressure in the ventricular chamber; -dp/dt_max_, maximal rate of the decline in blood pressure in the ventricular chamber; LVEDP, left ventricular end diastolic pressure; LVSP, left ventricular systolic pressure. (C) The levels of plasma BNP. The data are presented as the means ± SD. **P<0.01 vs. SO group; ^#^P<0.05, ^##^P<0.01 vs. MI group.

To investigate the effect of ghrelin on HF, a rat model of HF after MI was established, and the morphological changes in the different groups were examined. Eight weeks after LAD ligation, the hearts from rats in the MI group presented with abnormal, enlarged ventricular cavities. H&E staining showed that the cardiac myocytes exhibited an irregular shape and arrangement with myocardial fibrosis. Moreover, the number of cardiac myocytes was greatly reduced. Notably, 4 weeks of ghrelin treatment (MI-ghrelin group) significantly suppressed the signs of HF in the MI animals (Fig. 2A).

The hemodynamic assessment showed that the untreated model group (MI group) had increased LVEDP as well as decreased LVSP and ± dp/dt_max_. However, ghrelin administration significantly reduced the LVEDP levels and elevated the LVSP and ± dp/dt_max_ levels compared with the untreated model (P<0.05) (Fig. 2B).

BNP is another marker of HF. This study showed that the serum BNP level was promoted in the MI group compared with the sham operation group. However, BNP was significantly reduced in the ghrelin administration group (P<0.01) (Fig. 2C).These observations suggested that ghrelin protected against HF in rats with MI.

### Ghrelin inhibited cardiomyocyte apoptosis both in vivo and in vitro

**Figure 3:**
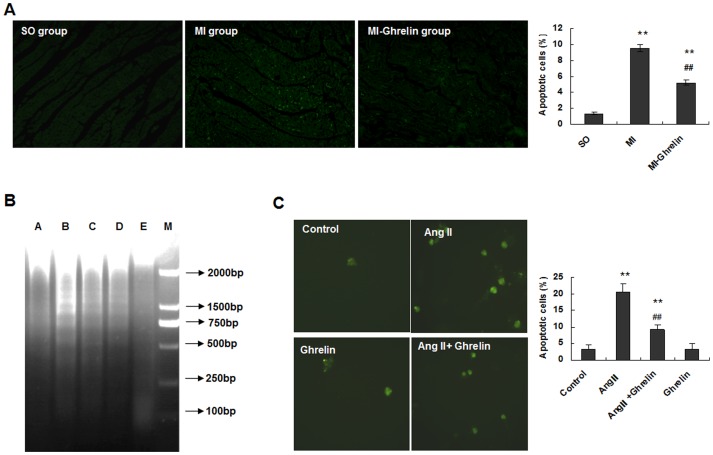
Ghrelin inhibited cardiomyocyte apoptosis both in vivo and in vitro. (A) TUNEL analysis was performed after the end of the ghrelin treatment. The TUNEL-positive cells (apoptotic cells) are indicated by arrows. The data are presented as the means ± SD. **P<0.01 vs. SO group, ^##^P<0.01 vs. MI group. (B) Ang II-induced DNA fragmentation in cardiomyocytes with or without ghrelin. Cultured cardiomyocytes from neonatal rats were stimulated with or without Ang II and ghrelin for 24 hours. The cardiomyocyte lysate was first incubated with RNase and then with proteinase K. By this method, only fragmented DNA was extracted. The DNA was separated by electrophoresis on a 1.5% agarose gel and stained with ethidium bromide. Lane A, vehicle-treated cardiomyocytes; lane B, cardiomyocytes incubated with 0.1 µmol/L Ang II; lane C, cardiomyocytes incubated with 1 µmol/L Ang II; lane D, cardiomyocytes incubated with 0.1 µmol/L ghrelin and 0.1 µmol/L Ang II; lane E, cardiomyocytes incubated with 0.1 µmol/L ghrelin. (C) Apoptosis of cardiomyocytes treated with Ang II and ghrelin. Cardiomyocytes were incubated in culture medium (control), 0.1 µmol/L Ang II, 0.1 µmol/L ghrelin, or 0.1 µmol/L ghrelin + 0.1 µmol/L Ang II for 24 hours. Magnification: ×200. The graph presents the percentage of TUNEL-positive cells determined from 100 cells in 3 independent experiments. The data are presented as the means ± SD. **P<0.01 vs. the control group;^ ##^P<0.01 vs. the Ang II group.

Cell apoptosis is one of the major outcomes of HF after MI, which indicates the molecular mechanism by which HF causes death. The observations that ghrelin protected against HF in rats with MI motivated us to further investigate the impacts of ghrelin on myocardial cell apoptosis. Apoptotic cells were analyzed by TUNEL staining, and the results showed that an increased number of apoptotic myocardial cells were present in rats with MI induced by LAD ligation whereas the ghrelin treatment dramatically decreased cell apoptosis (Fig. 3A).

To further confirm this outcome, cardiomyocytes were isolated from the cardiac ventricles of neonatal Wistar rats and cultured by differential adhesion to evaluate the effects of ghrelin. Twenty-four hours after Ang II stimulation, elevated numbers of apoptotic cells labeled by DNA Ladder were detected. Ghrelin greatly suppressed cell apoptosis induced by Ang II (Fig. 3B).

The apoptotic cells were further examined by TUNEL staining, and the results showed that the percentage of TUNEL-positive cells was significantly increased in the 0.1 µmol/L Ang II group compared with the control group (P<0.01). However, the percentage of TUNEL-positive cells in the 0.1 µmol/L Ang II + 0.1 µmol/L ghrelin group was significantly decreased compared with the 0.1 µmol/L Ang II group (P<0.01). There was no difference between the 0.1 µmol/L ghrelin group and the control group (P>0.05, Fig. 3C). Altogether, these results demonstrated that ghrelin inhibited cardiomyocyte apoptosis in HF both in vivo and in vitro and could prevent HF by inhibiting Ang II-induced cardiomyocyte apoptosis.

### Ghrelin mediated myocardium protection by caspase-3 down-regulation

**Figure 4:**
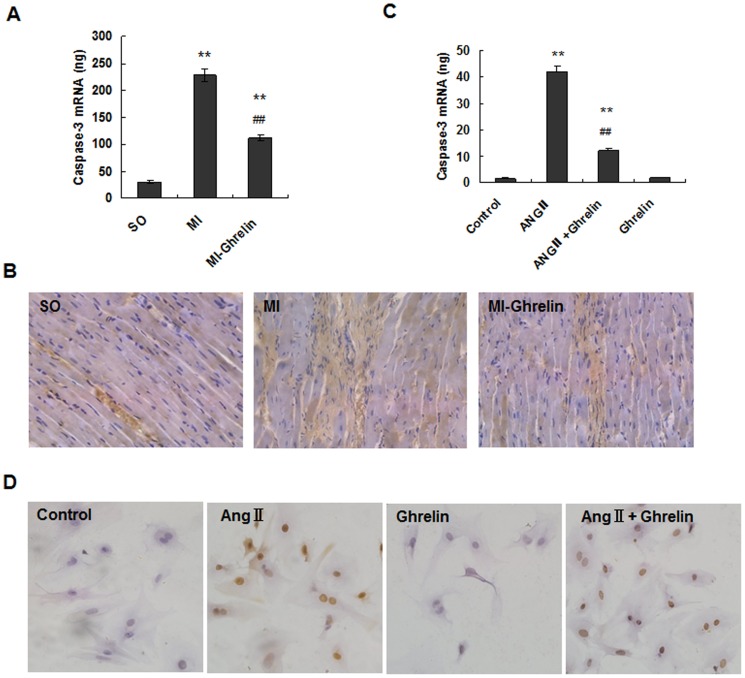
Ghrelin mediated myocardium protection by down-regulating caspase-3. (A) The expression of caspase-3 mRNA by real-time quantitative PCR. The total RNA of noninfarcted left ventricular tissue was extracted using TRIzol Reagent, and real-time quantitative PCR was performed to determine the caspase-3 mRNA levels. The data are presented as the means ± SD. **P<0.01 vs. SO group, ^##^P<0.01 vs. MI group. (B) The expression of caspase-3 in rat cardiac tissues examined by immunohistochemical staining (×200). (C) Analysis of caspase-3 mRNA levels in cardiomyocytes by quantitative real-time RT-PCR. After the addition of 0.1 µmol/L ghrelin, 0.1 µmol/L Ang II, 0.1 µmol/L ghrelin + 0.1 µmol/L Ang II, or culture medium (control), the cardiomyocytes were cultured for 24 hours. Real-time quantitative PCR was performed to determine the mRNA levels. The data are presented as the means ± SD. **P<0.01 vs. control group; ^##^P<0.01 vs. Ang II group. (D) Detection of caspase-3 protein expression in rat cardiomyocytes by immunocytochemical staining. After the addition of 0.1 µmol/L ghrelin, 0.1 µmol/L Ang II, 0.1 µmol/L ghrelin + 0.1 µmol/L Ang II, or culture medium (control), the cardiomyocytes were cultured for 24 hours. Magnification: ×200.

Caspase-3 is a key molecule in the apoptosis pathway, and its expression is positively correlated with cell apoptosis. To establish the mechanism of cell apoptosis inhibition induced by ghrelin, caspase-3 was detected using quantitative real-time PCR and immunocytochemistry in vivo. The results all showed that compared with the sham operation (SO) group, caspase-3 expression was significantly increased in the MI group (P<0.01). However, the ghrelin treatment significantly decreased caspase-3 expression (P<0.01) (Fig. 4A, B).

Furthermore, we examined caspase-3 expression in cardiomyocytes stimulated with Ang II and/or ghrelin using quantitative real-time PCR and immunocytochemistry in vitro. The results all showed that the caspase-3 levels were significantly increased in the Ang II group compared with those in the control group (P<0.01). However, the caspase-3 levels were significantly lower in the Ang II +ghrelin group than in the Ang II group (P<0.01) (Fig. 4C, D). These results indicated that Ang II induces cardiomyocyte apoptosis through the caspase-3 pathway whereas ghrelin inhibits this action.

### Ghrelin inhibits Ang II-induced cell apoptosis by down-regulating AT1R, thereby protecting against HF

**Figure 5:**
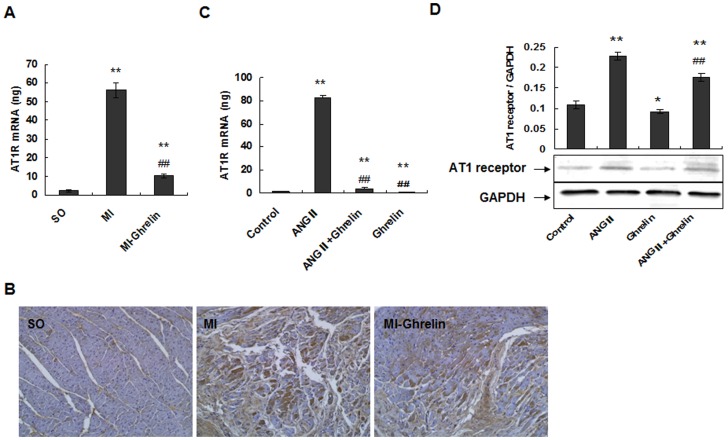
Ghrelin inhibits Ang II-induced cell apoptosis by down-regulating AT1R and thereby preventing HF. (A) The expression of AT1R mRNA by real-time quantitative PCR. The total RNA of noninfarcted left ventricular tissue was extracted using TRIzol Reagent, and real-time quantitative PCR was performed to determine the AT1R mRNA levels. The data are presented as the means ± SD. **P<0.01 vs. SO group, ^##^P<0.01 vs. MI group. (B) The expression of cardiac AT1R in rat cardiac tissues by immunohistochemical staining (×200). (C) Real-time quantitative PCR was performed to determine the AT1 receptor mRNA levels in cardiomyocytes. After the addition of 0.1 µmol/L ghrelin, 0.1 µmol/L Ang II, 0.1 µmol/L ghrelin + 0.1 µmol/L Ang II, or culture medium (control), the cardiomyocytes were cultured for 24 hours. Real-time quantitative PCR was performed to determine the mRNA levels. The data are presented as the means ± SD. **P<0.01 vs. the control group; ^##^P<0.01 vs. the Ang II group. (D) Western blotting was performed to determine AT1 receptor expression. Proteins were extracted from cardiomyocytes, separated by SDS-PAGE, and immunoblotted sequentially with anti-AT1 receptor antibody. The graph shows the result of densitometric quantification of the AT1 receptor protein relative to GAPDH as an internal control. The data are presented as the means ± SD. *P<0.05, **P<0.01 vs. the control group; ^##^P<0.01 vs. the Ang II group.

To further elucidate the mechanism responsible for the inhibition of Ang II action by ghrelin, the expression of the AT1 receptors was evaluated using quantitative real-time PCR and immunocytochemistry in vivo. The results all showed that compared with the SO group, AT1 receptor expression was significantly increased in the MI group (P<0.01). However, the ghrelin treatment significantly decreased the AT1 receptor expression (P<0.01) (Fig. 5A, B).

To confirm this observation, we detected the expression of the AT1 receptors using quantitative real-time PCR and western blotting in vitro. The quantitative real-time PCR results showed that AT1 receptor mRNA expression significantly increased in the Ang II group compared with the control group (P<0.01). The expression of AT1 receptor mRNA also increased in the Ang II+ghrelin treatment group (P<0.01) but remained significantly lower than that in the Ang II group (P<0.01). Additionally, AT1 receptor mRNA expression was significantly decreased in the ghrelin group compared with the control group (P<0.01) (Fig. 5C). Western blot analyses were consistent with the above results. AT1 receptor protein expression was significantly increased in the Ang II group compared with the control group (P<0.01); AT1 receptor expression in the Ang II+ghrelin group was also increased compared with the control group (P<0.01) but significantly decreased compared with the Ang II group (P<0.01) (Fig. 5D). These results suggest that Ang II may bind to the AT1 receptor and induce cardiomyocyte apoptosis, whereas ghrelin inhibits cardiomyocyte apoptosis by blocking AT1 receptor up-regulation.

**Figure 6:**
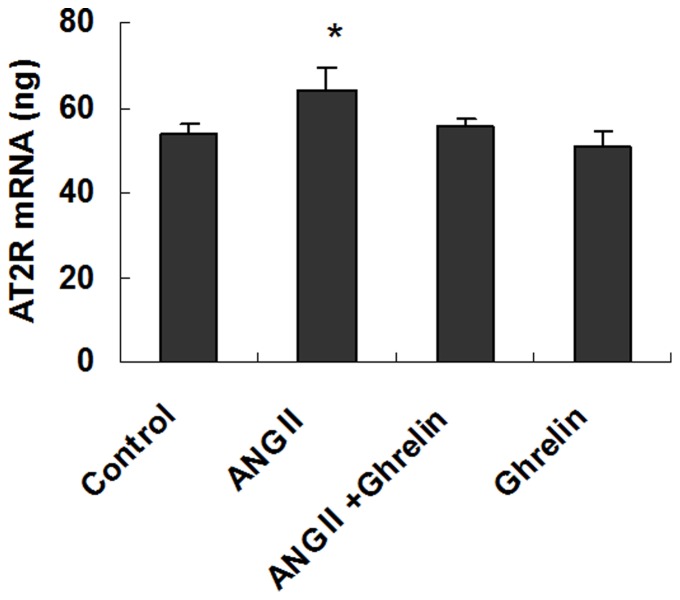
Real-time quantitative PCR was performed to determine the AT2 receptor mRNA levels. After the addition of 0.1 µmol/L ghrelin, 0.1 µmol/L Ang II, 0.1 µmol/L ghrelin + 0.1 µmol/L Ang II, or culture medium (control), the cardiomyocytes were cultured for 24 hours. Real-time quantitative PCR was performed to determine the mRNA levels. The data are presented as the means ± SD. *P<0.05 vs. the control group.

In addition, we detected the expression of the AT2 receptors using quantitative real-time PCR. The expression of AT2 receptor mRNA was also increased in the Ang II group compared with the control group (P<0.05), but the expression of the AT2 receptor mRNA in the Ang II+ghrelin group was not significantly different from that in the Ang II group. Additionally, the expression levels of AT2 receptor mRNA in the Ang II+ghrelin group, ghrelin group and control group were not different (P>0.05) (Fig. 6). These results indicate that Ang II can up-regulate the AT1 and AT2 receptors in cardiomyocytes but that ghrelin inhibits the up-regulation of AT1 receptor but does not affect AT2 receptor expression.

## Discussion

Ghrelin, a 28-amino acid peptide, was first reported by Kojima in rat and human stomachs in 1999 [17]. Some studies have reported that ghrelin confers a variety of potentially beneficial cardiovascular effects, which include reducing blood pressure, increasing cardiac contractility, protecting endothelial cells, regulating atherosclerosis, and improving the prognoses of MI and HF [19]–[22]. However, the exact mechanism by which ghrelin confers these effects remains unclear. Ang II, a key molecule in the renin-angiotensin-aldosterone system (RAAS), is significantly increased during HF. The increase in the Ang II level can improve cardiac function by constricting the arteries to increase blood pressure, thereby regulating blood volume in the short term. However, because the increase in the Ang II level can lead to hypertension, collagen synthesis, and myocyte apoptosis, long-lasting increased Ang II levels damage the heart, leading to ventricular remodeling and HF [24].

In the present study, we found that the serum levels of Ang II and ghrelin are both significantly increased in patients with HF and that serum ghrelin levels are closely related to increases in the Ang II level. As Ukkola and colleagues have verified that the highest plasma ghrelin tertile was associated with increased intraventricular septum and posterior ventricular wall thicknesses as well as left ventricular mass [25], we consider that the increased serum ghrelin levels in HF may be a compensatory protective reaction to the damage caused by long-lasting increased Ang II levels. The increased serum ghrelin level in HF is likely to prevent HF by inhibiting Ang II.

To further explore the potential significance of serum ghrelin level changes in HF, we established the post-MI model of HF in rats and administered a ghrelin intervention. The results demonstrated that ghrelin can improve cardiac function in CHF rats, as indicated by increases in LVSP and LV dp/dt_max_ and by decreases in LVEDP, left ventricular mass to body weight (LVM/BW), and the number of apoptotic cardiomyocytes. These results are consistent with previous findings that ghrelin prevents early left ventricular remodeling in rats with MI [26], [27]. We further clarified that increased serum ghrelin levels are a protective response in HF, which can improve cardiac function and improve the HF prognosis.

The progressive deterioration of the hypertrophied left ventricle (LV), which eventually precipitates into HF, is generally accepted to be related to the progressive loss of cardiac myocytes [5], [6]. In the present study, we found that the number of apoptotic cells in the MI-ghrelin treated animals was significantly lower than that of the MI group. The above results suggest that ghrelin can improve cardiac function by inhibiting cardiomyocyte apoptosis. Moreover, using in vitro experiments, we also found that ghrelin can inhibit Ang II-induced cardiomyocyte apoptosis, which further validated that ghrelin has a cardiovascular protective effect via Ang II inhibition.

Caspase-3 is a key factor in the apoptotic pathway, and caspase-3 expression is positively correlated with apoptosis [28], [29]. In this study, in vivo and vitro experimental results both showed that ghrelin inhibited cardiomyocyte apoptosis by down-regulating caspase-3 expression. These results further suggest that ghrelin plays an anti-HF role, which may be achieved by inhibiting the Ang II-induced caspase-3 up-regulation of cell apoptosis.

The effect of Ang II on cardiomyocytes is related to the activation of specific receptors. Ang II receptors belong to the large family of 7 transmembrane receptors and have been divided into 2 pharmacologically distinct types, type 1 (AT1) and type 2 (AT2) [9], [10]. Although previous studies have shown that Ang II can induce cardiomyocyte apoptosis [30], controversy remains over which receptor mediates Ang II-induced cardiomyocyte apoptosis. Therefore, to clarify the roles of the AT1 and AT2 receptors in Ang II-induced cardiomyocyte apoptosis, we evaluated the expression of the AT1 and AT2 receptors. Our data showed that the expression of both the AT1 and AT2 receptors significantly increased in the Ang II group. However, in the Ang II+ghrelin treatment group, only the AT1 receptor was down-regulated, whereas the AT2 receptor expression was not changed. Additionally, AT1 receptor expression was significantly reduced in the ghrelin group compared with the control group, but the AT2 receptor expression in the ghrelin group was not significantly different from that of the control group. These data indicate that Ang II may bind mainly to the AT1 receptor and induce cardiomyocyte apoptosis and that ghrelin inhibits cardiomyocyte apoptosis via reducing the Ang II-up-regulation of the AT1 receptor.

In conclusion, the present study demonstrates that the serum levels of Ang II and ghrelin are both significantly increased in patients with HF and that increased ghrelin inhibits cardiomyocyte apoptosis via down-regulating AT1 receptor and caspase-3 expression. This outcome suggests that ghrelin may exert a cardio-protective effect against the role of Ang II in the treatment of HF. These mechanisms will provide the mechanical basis for the development of ghrelin as a clinical application to treat HF.
